# Multisite Head and Neck Pediatric Posttransplant Lymphoproliferative Disorder: A Case Report

**DOI:** 10.1002/ccr3.70200

**Published:** 2025-02-14

**Authors:** Lily Gao, Austin Hoke, Jarrett Jackson, Laura Petrauskas, Emily F. Mason, Asha Sarma, Debra Friedman, David Bearl, Daniel Dulek, Christopher Wootten, Jason Park

**Affiliations:** ^1^ Vanderbilt University School of Medicine Nashville Tennessee USA; ^2^ Department of Otolaryngology‐Head & Neck Surgery Vanderbilt University Medical Center Nashville Tennessee USA; ^3^ Department of Pathology, Microbiology and Immunology Vanderbilt University Medical Center Nashville Tennessee USA; ^4^ Department of Radiology Vanderbilt University Medical Center Nashville Tennessee USA; ^5^ Department of Pediatrics, Hematology and Oncology Vanderbilt Children's Hospital Nashville Tennessee USA; ^6^ Department of Pediatrics, Cardiology Vanderbilt Children's Hospital Nashville Tennessee USA; ^7^ Department of Pediatrics, Infectious Diseases Vanderbilt Children's Hospital Nashville Tennessee USA

**Keywords:** larynx, lymphoma, posttransplant lymphoproliferative disorder, sinonasal, sinusitis, skull base

## Abstract

Posttransplant lymphoproliferative disorder (PTLD) can mimic infectious processes in the head and neck. A high index of suspicion for PTLD must be maintained in pediatric transplant patients, and minimal response to antibiotics/corticosteroids should prompt timely biopsy of suspicious tissue to expedite the diagnosis and treatment of PTLD.

## Graphical abstract

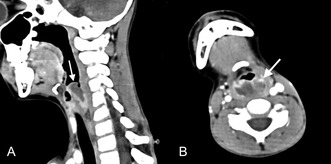

**Abbreviations**
ADC: apparent diffusion coefficient
AIFS: acute invasive fungal sinusitis
CT: computed tomography
DLBCL: diffuse large B‐cell lymphoma
EBER: Epstein–Barr encoding region
EBV: Epstein–Barr virus
ENT: ear, nose, throat
GI: gastrointestinal
H&E: hematoxylin and eosin
IU: international units
mL: milliliter
MRI: magnetic resonance imaging
PAX5: paired box protein 5
PTLD: post‐transplant lymphoproliferative disorder

## 1 Introduction

Posttransplant lymphoproliferative disorder (PTLD) is the most common malignancy in pediatric transplant patients [1]. PTLD is often associated with Epstein–Barr virus (EBV) infection, which induces unchecked B lymphocyte proliferation in the setting of chronic immunosuppression [1]. Risk factors for developing PTLD include negative recipient pretransplant EBV serology and being within 1 year of transplantation [1, 2, 3]. PTLD is diagnosed with tissue biopsy and is classified into histologic subtypes, including benign proliferative, polymorphic, monomorphic, and classic Hodgkin's lymphoma PTLD [4]. Polymorphic PTLD is the most common subtype in children and is characterized by a heterogenous mixture of atypical lymphoid cells, while monomorphic PTLD is characterized by aggressive, clonal expansion of cells, with the majority of monomorphic PTLDs being categorized as diffuse large B‐cell lymphoma (DLBCL) [5, 6]. Polymorphic PTLD is typically treated with reduction of immunosuppression and rituximab, while monomorphic PTLD is treated similarly but with the addition of chemotherapy [7, 8].

In pediatric PTLD patients, 25%–63% have head and neck involvement, which may present as airway obstruction due to lymphoid hypertrophy [9]. PTLD involvement of the laryngotracheal airway and paranasal sinuses is rare [10, 11]. We present a unique case of PTLD that mimicked infectious processes in the head and neck and ultimately required multiple tissue biopsies to diagnose its involvement of the cervical lymph nodes, supraglottis, and paranasal sinuses with intracranial extension.

## 2 Case Presentation

A 7‐year‐old boy with negative EBV serology underwent orthotopic heart transplant for Ebstein's anomaly. His transplant immunosuppressive regimen included mycophenolate mofetil induction therapy followed by tacrolimus and sirolimus maintenance therapy. Four months posttransplant, he was admitted to the hospital for odynophagia and fever, testing positive for streptococcal pharyngitis and EBV (plasma PCR 7270 IU/mL). A CT scan of the neck with contrast showed enlargement and striated enhancement of the palatine tonsils and enlarged cervical lymph nodes with central hypoenhancement, which were thought to represent tonsillitis and suppurative lymphadenitis. The patient received a short course of intravenous antibiotics during inpatient observation and defervesced appropriately. He was discharged without changes to his immunosuppressive regimen (4 months post transplantation).

Two weeks later, he was readmitted with fever and new stertorous breathing. His EBV load had increased from the recent admission (9810 IU/mL). Flexible nasolaryngoscopy demonstrated adenotonsillar hypertrophy without overt mass lesions. CT imaging showed an interval decrease in the size of his adenotonsillar hypertrophy and cervical lymphadenopathy compared to prior cross‐sectional imaging. Given his interval radiographic improvement, no surgical intervention was recommended. The patient's immunosuppressive regimen was reduced to tacrolimus monotherapy to aid in the resolution of the EBV infection. He was again discharged home in stable condition (5 months post transplantation).

Four days after discharge, the patient was readmitted for lethargy and odynophagia and was found to have a markedly elevated EBV viral load of 284,000 IU/mL. CT of the neck with contrast showed increased adenotonsillar enlargement and diffuse cervical adenopathy.

## 3 Differential Diagnosis, Investigations, and Treatment

The differential diagnosis for this immunocompromised patient's fever, odynophagia, and lymphoid hypertrophy initially included infectious causes such as tonsillitis and pharyngitis secondary to Group A Streptococci, EBV, or other opportunistic pathogens; however, the combination of his worsening imaging findings, persistent symptoms despite antibiotics/anti‐inflammatory therapies, and significant increase in EBV viral load raised concern for neoplastic etiologies such as posttransplant lymphoproliferative disorder.

Given the increased concern for PTLD, the patient's progressive airway symptoms, and his hemodynamic stability, a collective decision was made to perform adenotonsillectomy and excisional cervical lymph node biopsy for diagnostic and therapeutic purposes. Polymorphic PTLD was identified in the cervical lymph node (Figure S1) (5 months post transplantation). Subsequently, the patient was initiated on 4 weeks of rituximab infusions and remained on his reduced immunosuppressive regimen of tacrolimus monotherapy.

**FIGURE 1:**
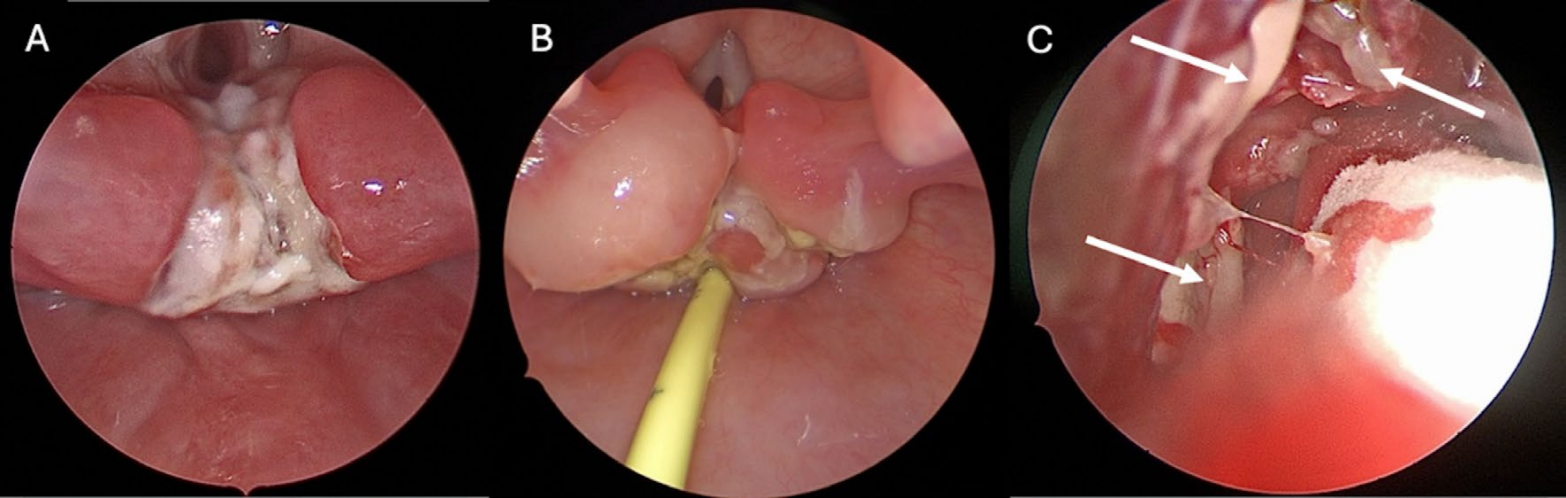
Intraoperative findings. (A) Direct laryngoscopy performed at 6 months posttransplant showed friable, sloughing epithelial debris and friable mucosa primarily involving the interarytenoid region. Biopsy of this supraglottic lesion demonstrated a mixed inflammatory infiltrate with necrosis and EBV‐positive B cells of uncertain significance. (B) Direct laryngoscopy performed at 8 months posttransplant revealed progression of the previously biopsied supraglottic lesion, marked arytenoid edema, and fibrinous plaques extending from the interarytenoid region to the post‐cricoid mucosa/cartilage and esophageal inlet. Biopsy of this supraglottic lesion demonstrated monomorphic posttransplant lymphoproliferative disorder (PTLD) presenting as EBV‐positive diffuse large B‐cell lymphoma (DLBCL). (C) Nasal endoscopy performed at 9 months posttransplant revealed white/yellow necrotic mass‐like lesions (arrows) without viable bleeding in the right ethmoid cavity. Biopsy of lesions in the right sphenoethmoidal recess and ethmoid air cells demonstrated monomorphic PTLD presenting as EBV‐positive DLBCL.

Two weeks after the initiation of rituximab, the patient was readmitted with fevers, odynophagia, and emesis. CT scans demonstrated new heterogeneous hypopharyngeal enhancement, and operative direct laryngoscopy showed sloughing epithelial debris and plaques in the interarytenoid region (Figure 1A). Biopsy was performed due to concern for new PTLD involvement, which revealed necrosis and scattered EBV‐positive B cells of uncertain significance. The patient was discharged in afebrile, stable condition on antibiotics and antifungals for presumed infectious laryngitis (5.5 months post transplantation).

After completing 4 weeks of rituximab, the patient was clinically stable with a low EBV load (400 IU/mL). He remained on tacrolimus monotherapy (6 months post transplantation).

**FIGURE 2:**
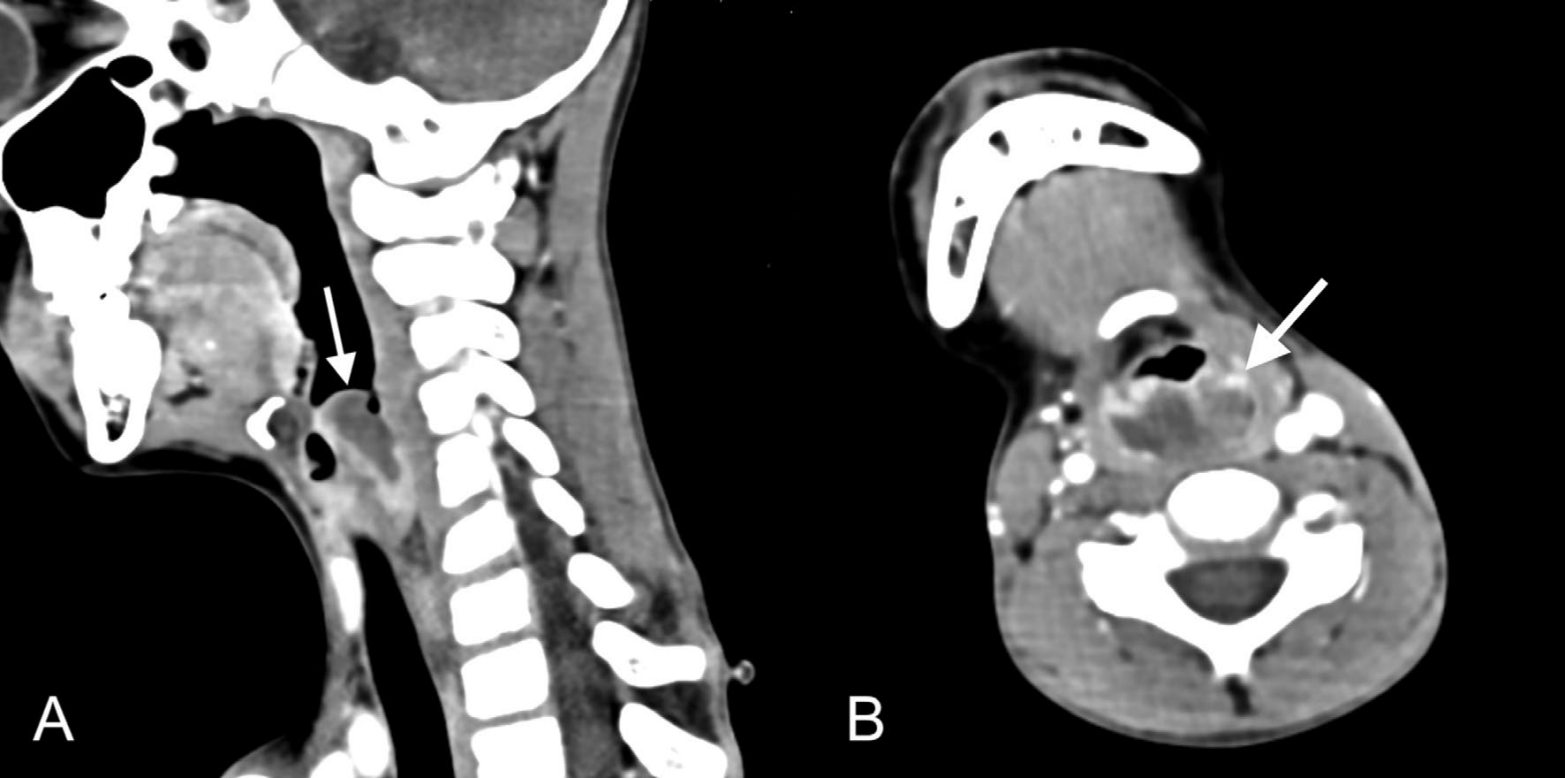
Contrast‐enhanced CT neck showing a supraglottic lesion, 8 months posttransplant. Sagittal (A) and axial (B) images show a heterogeneous lesion (arrows) with central hypoattenuation and peripheral irregular enhancement involving the aryepiglottic folds, with narrowing of the supraglottic airway. Biopsy of the lesion demonstrated monomorphic posttransplant lymphoproliferative disorder presenting as EBV‐positive diffuse large B‐cell lymphoma.

Two months later, the patient was readmitted for fever, diarrhea, and weight loss. CT imaging showed a heterogeneously enhancing supraglottic mass with airway narrowing (Figure 2), pulmonary nodules, and intestinal wall thickening. ENT, pulmonology, and GI services performed a joint endoscopic evaluation. Direct laryngoscopy showed arytenoid ulceration and plaques extending from the posterior cricoid space to the esophageal inlet (Figure 1B). Biopsies of the hypopharynx, supraglottis, stomach, and small bowel demonstrated monomorphic PTLD presenting as EBV‐positive DLBCL (Figure S2) (8 months post transplantation). The following week, the patient underwent small bowel resection for bowel obstruction. Multiple perforations and areas of necrosis related to PTLD were identified intraoperatively.

## 4 Outcome & Follow up

**FIGURE 3:**
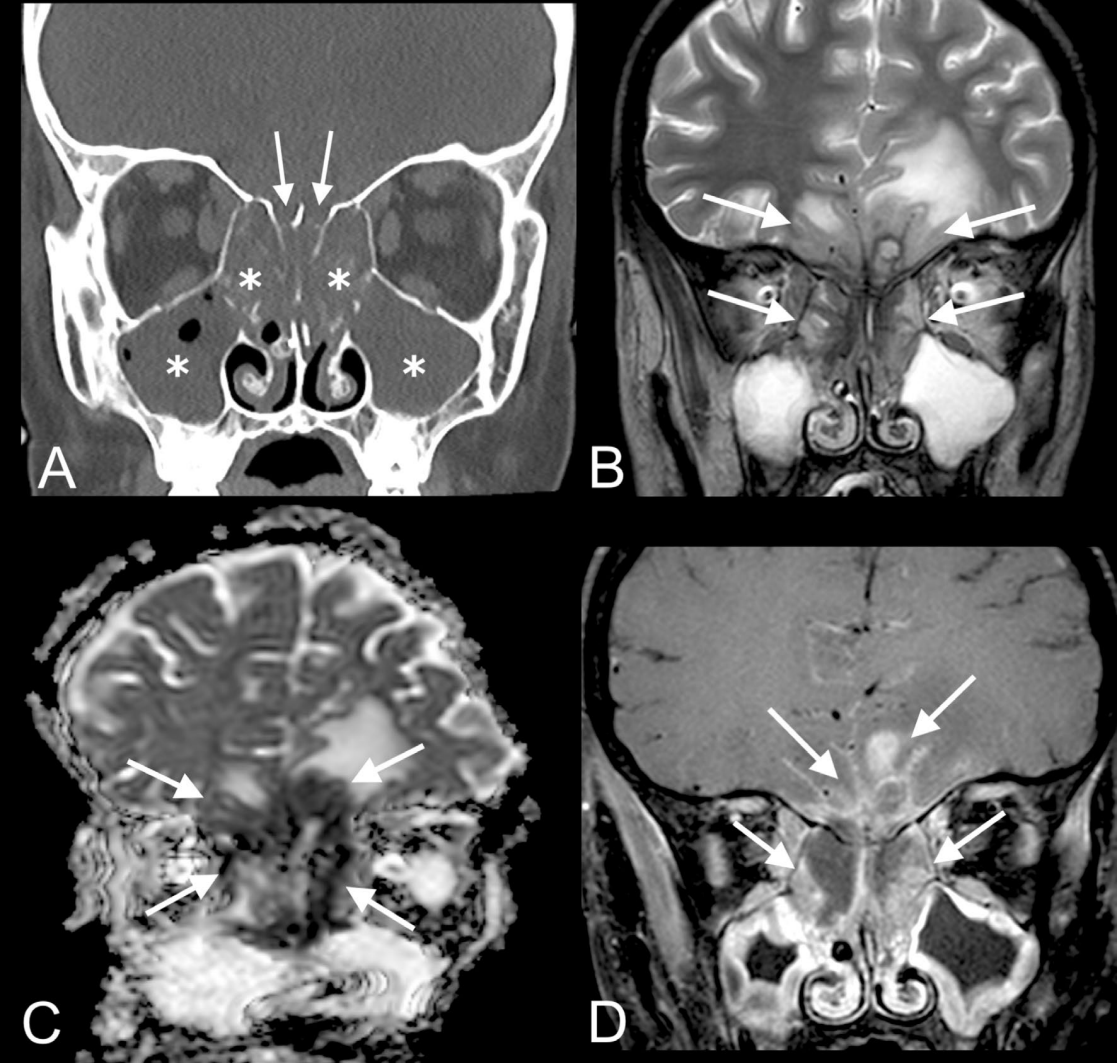
CT and MRI images of the paranasal sinuses, 9 months posttransplant. Coronal CT sinus (A) shows diffuse opacification of the maxillary and ethmoid sinuses (asterisks) with erosion of the ethmoid septations and cribriform plates (arrows). Coronal T2‐weighted brain MRI (B) shows mixed hypo‐ and hyperintense lesions (arrows) within the ethmoid sinuses that extend through the cribriform plates into the inferior frontal lobes, greater on the left than on the right, with adjacent vasogenic edema. The lesions demonstrate corresponding restricted diffusion and enhancement on coronal ADC map (C) and coronal post‐contrast fat‐suppressed T1‐weighted imaging (D), respectively. The differential diagnosis included posttransplant lymphoproliferative disorder (PTLD) and acute invasive fungal sinusitis. Biopsy of the lesion in the right sphenoethmoidal recess demonstrated monomorphic PTLD presenting as EBV‐positive diffuse large B‐cell lymphoma.

**FIGURE 4:**
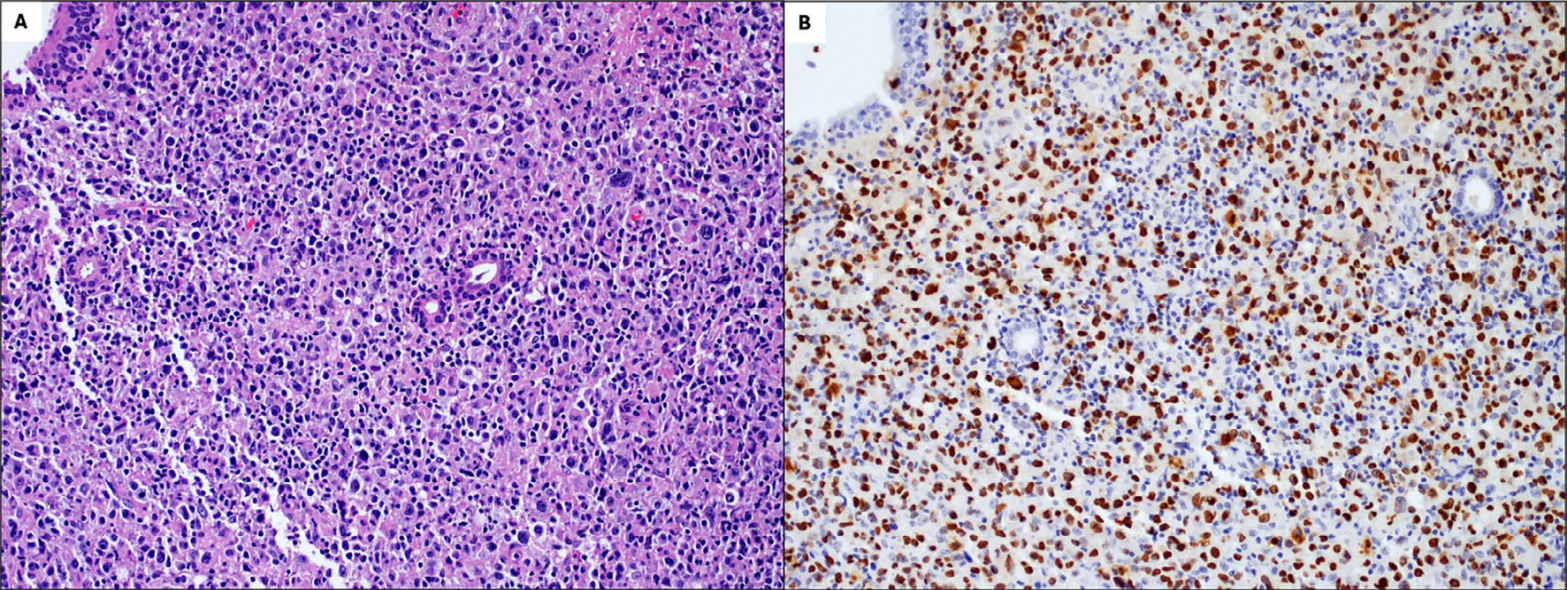
Monomorphic post‐transplant lymphoproliferative disorder presenting as EBV‐positive diffuse large B‐cell lymphoma. Biopsy of a necrotic mass in the right sphenoethmoidal recess at 9 months posttransplant showed a diffuse infiltrate of large lymphocytes with prominent nuclei on H&E stain (A). The large cells were PAX5‐positive B cells (not shown) that were diffusely positive for EBV by EBV‐encoded RNA (EBER) in situ hybridization (B). All images 200×.

Treatment for monomorphic PTLD was initiated postoperatively and included rituximab and combination chemotherapy. The patient continued to decline clinically, and a brain MRI obtained in the setting of altered mental status 1 month later showed abnormal enhancement of the paranasal sinuses extending intracranially into the inferomedial frontal lobes (Figure 3). A CT sinus showed pan‐sinus opacification with erosion of the cribriform plate and ethmoid septations, which was concerning for advanced PTLD versus acute invasive fungal sinusitis (AIFS) (Figure 3). Operative nasal endoscopy was performed (Figure 1C), and a biopsy of a necrotic mass in the right sphenoethmoidal recess revealed monomorphic PTLD presenting as EBV‐positive DLBCL (Figure 4) (9 months post transplantation).

Ultimately, given the extensive involvement of PTLD and the patient's multiorgan involvement, the patient was transitioned to comfort care and expired (6 weeks after final admission, 9 months post transplantation).

## 5 Discussion

PTLD affects 5%–10% of pediatric heart transplant patients [2, 12]. This case of PTLD ultimately involved the GI tract, lungs, and multiple sites in the head and neck, including cervical lymph nodes, the larynx, and paranasal sinuses, with intracranial extension. PTLD involvement of adenotonsillar and nodal lymphoid tissue is common, but PTLD lesions in the larynx and sinuses may represent particularly aggressive clinical entities, as evidenced by the widespread PTLD lesions that developed despite escalating treatment with reduction of immunosuppression, rituximab, and chemotherapy [9, 10]. Throughout the course of this patient's illness, PTLD was challenging to distinguish from various infectious processes of the head and neck. Biopsies were pursued after minimal response to antimicrobial therapy and were critical for the diagnoses of polymorphic and monomorphic PTLD.

First, PTLD involvement of the cervical lymph nodes presented with fever, lymphadenopathy, and tonsillar hypertrophy, which mimicked tonsillitis secondary to streptococcal and/or EBV infection. Moreover, the patient had positive diagnostic testing for these microbial infections at the time of his initial presentation. Polymorphic PTLD was diagnosed by surgical biopsy 2 weeks after the initial presentation. This biopsy was pursued in the setting of minimal improvement following antibiotics and highlights the importance of sampling abnormal lymphoid tissue in a timely manner to diagnose PTLD and begin treatment, which in this case included maintaining a reduced immunosuppressive regimen and initiating rituximab.

Next, PTLD involvement of the hypopharynx and supraglottis presented as airway obstruction and exhibited ulcerations and plaques on laryngoscopy, which mimicked infectious laryngitis. Two prior cases of pediatric laryngeal PTLD presented similarly with airway obstruction, progressive stridor, and infiltrates in the arytenoids and epiglottis [13]. This patient's supraglottic lesion was ultimately diagnosed as monomorphic PTLD 2 months after a prior negative surgical biopsy. Repeat biopsy was pursued in the setting of worsening symptoms despite completing antibiotic and antifungal therapy as well as the initial treatment for polymorphic PTLD. This diagnosis of monomorphic PTLD found on repeat biopsy highlights the importance of maintaining a high index of suspicion for new PTLD lesions in a patient with a history of PTLD, even after an initial negative biopsy in an atypical location, because recurrence or transformation to monomorphic PTLD remains a possibility and would require additional treatment.

Lastly, PTLD involvement of the paranasal sinuses and anterior intracranial compartment was challenging to differentiate from acute invasive fungal sinusitis on clinical presentation and imaging. Two previous cases of adult sinonasal PTLD also proved challenging to distinguish from AIFS, which can present with altered mental status and intracranial involvement in immunocompromised patients [11, 14]. Hatten et al. noted that the timeline of symptoms may help distinguish between the two entities, with AIFS typically developing within days and PTLD developing over weeks to months, but timing was less informative in this case due to the patient being intubated and sedated in the weeks prior to diagnosis [11]. PTLD can also mimic AIFS radiographically. The fungal elements of AIFS characteristically display T2 hypointense components with loss of enhancement of well‐vascularized structures such as the nasal mucosa. Similarly, PTLD may also display T2 hypointense signal and restricted diffusion due to hypercellularity. A biopsy of this patient's sinus mass ultimately demonstrated monomorphic PTLD and highlights that in patients with preexisting PTLD, new lesions are still likely to represent PTLD despite atypical locations and ongoing treatment for PTLD.

This study is limited by its design as a single‐patient case report. As a result, this study may not be generalizable and may also have biases that are inherent to its retrospective design. Additionally, anecdotal reporting of this patient's multifocal disease involvement does not offer sufficient evidence to support the identification of a novel, distinct pathologic process.

## Author Contributions

**Lily Gao:** writing – original draft, writing – review and editing. **Austin Hoke:** writing – original draft, writing – review and editing. **Jarrett Jackson:** writing – original draft. **Laura Petrauskas:** writing – original draft. **Emily F. Mason:** writing – review and editing. **Asha Sarma:** writing – review and editing. **Debra Friedman:** writing – review and editing. **David Bearl:** writing – review and editing. **Daniel Dulek:** writing – review and editing. **Christopher Wootten:** supervision, writing – review and editing. **Jason Park:** supervision, writing – review and editing.

## Consent

Written consent was obtained from the mother of the patient after death.

## Conflicts of Interest

The authors declare no conflicts of interest.

## Supporting information


**Figure S1.** Polymorphic posttransplant lymphoproliferative disorder. (A) Low power (40×) and (B) high power (400×) images show effacement of nodal architecture by a mixed inflammatory infiltrate composed of small to intermediate‐sized lymphocytes, plasma cells, and immunoblasts. Immunohistochemical stains show a mixture of CD3‐positive T cells (C), CD20‐positive B cells (D), and CD138‐positive plasma cells (E). In situ hybridization for EBV‐encoded RNA (EBER) was diffusely positive (F). Plasma cells and plasmacytoid B cells show focal areas with excess kappa light chain expression (G) and other areas with excess lambda light chain expression (H), with polytypic light chain expression overall by kappa/lambda in situ hybridization. All immunostain images 200×.

**Figure S2.** Monomorphic posttransplant lymphoproliferative disorder presenting as EBV‐positive diffuse large B cell lymphoma. (A–C) Biopsies of an umbilicated gastric mass showed a diffuse infiltrate of large lymphocytes with prominent nuclei on H&E stain (A). The large cells were CD20‐positive B cells (B) that were diffusely positive for EBV by EBER in situ hybridization (C). (D–F) Biopsy of the ethmoid sinus showed similar findings on H&E stain (D), with a diffuse infiltrate of large lymphoid cells, which were positive for PAX5 (E) and diffusely positive for EBER (F). (A–C) All images 400×. (D–F) All images 200×.

## Data Availability

Data sharing is not applicable to this article as no datasets were generated or analyzed during the study.

## References

[1] E. Marie , M. Navallas , O. M. Navarro , et al., “Posttransplant Lymphoproliferative Disorder in Children: A 360‐Degree Perspective,” Radiographics 40, no. 1 (2020): 241–265, 10.1148/rg.2020190103. 31834850

[2] I. C. Kim , S. H. Kim , J. C. Youn , et al., “Temporal Trends, Risk Factors, and Clinical Outcomes of De Novo Lymphoproliferative Disorders After Heart Transplantation,” JACC: Heart Failure 12, no. 2 (2024): 395–405, 10.1016/j.jchf.2023.10.018. 38326002

[3] U. T. Offor , C. M. Bacon , J. Roberts , et al., “Transplantation for Congenital Heart Disease Is Associated With an Increased Risk of Epstein‐Barr Virus–Related Post‐Transplant Lymphoproliferative Disorder in Children,” Journal of Heart and Lung Transplantation 40, no. 1 (2021): 24–32, 10.1016/j.healun.2020.10.006. 33339556

[4] S. H. Swerdlow , E. Campo , S. A. Pileri , et al., “The 2016 Revision of the World Health Organization Classification of Lymphoid Neoplasms,” Blood 127, no. 20 (2016): 2375–2390, 10.1182/blood-2016-01-643569. 26980727 PMC4874220

[5] G. Végso , M. Hajdu , and A. Sebestyén , “Lymphoproliferative Disorders After Solid Organ Transplantation‐Classification, Incidence, Risk Factors, Early Detection and Treatment Options,” Pathology & Oncology Research 17, no. 3 (2011): 443–454, 10.1007/s12253-010-9329-8. 21193979

[6] Y. Liu , B. C. Wang , C. W. Zuppan , et al., “Relationship of Post‐Transplant Lymphoproliferative Disorders (PTLD) Subtypes and Clinical Outcome in Pediatric Heart Transplant Recipients: A Retrospective Single Institutional Analysis/Experience of 558 Patients,” Cancers 15, no. 3 (2023): 976, 10.3390/cancers15030976. 36765933 PMC9913467

[7] A. Parker , K. Bowles , J. A. Bradley , et al., “Management of Post‐Transplant Lymphoproliferative Disorder in Adult Solid Organ Transplant Recipients – BCSH and BTS Guidelines,” British Journal of Haematology 149, no. 5 (2010): 693–705, 10.1111/j.1365-2141.2010.08160.x. 20408848

[8] R. Asleh , H. Alnsasra , T. M. Habermann , A. Briasoulis , and S. S. Kushwaha , “Post‐Transplant Lymphoproliferative Disorder Following Cardiac Transplantation,” Frontiers in Cardiovascular Medicine 9 (2022): 787975, 10.3389/fcvm.2022.787975. 35282339 PMC8904724

[9] J. Roberts , J. Powell , M. W. Mather , S. Powell , and M. Brodlie , “A Review of Adenotonsillar Hypertrophy and Adenotonsillectomy in Children After Solid Organ Transplantation,” International Journal of Pediatric Otorhinolaryngology 114 (2018): 29–35, 10.1016/j.ijporl.2018.08.020. 30262363

[10] C. A. Banks , J. D. Meier , C. R. Stallworth , and D. R. White , “Recurrent Posttransplant Lymphoproliferative Disorder Involving the Larynx and Trachea: Case Report and Review of the Literature,” Annals of Otology, Rhinology, and Laryngology 121, no. 5 (2012): 291–295, 10.1177/000348941212100502. 22724273

[11] K. M. Hatten , L. A. Loevner , J. N. Palmer , and N. D. Adappa , “Isolated Sinonasal Posttransplantation Lymphoproliferative Disorder: A Clinical and Radiographic Invasive Fungal Sinusitis Look‐a‐Like,” ORL‐Journal For Oto‐Rhino‐Laryngology and its Related Specialties 74, no. 6 (2012): 339–342, 10.1159/000346240. 23392283

[12] S. A. Webber , D. C. Naftel , F. J. Fricker , et al., “Lymphoproliferative Disorders After Paediatric Heart Transplantation: A Multi‐Institutional Study,” Lancet 367, no. 9506 (2006): 233–239, 10.1016/S0140-6736(06)67933-6. 16427492

[13] N. D. Vandjelovic , P. C. Barth , S. P. Dunn , K. R. Chikwava , and U. K. Shah , “Post‐Transplant Lymphoproliferative Disease of the Larynx,” Journal of Surgical Case Reports 2019, no. 4 (2019): rjz111, 10.1093/jscr/rjz111. 30967940 PMC6451186

[14] A. R. Gordon , L. A. Loevner , A. I. Sonners , W. E. Bolger , and M. A. Wasik , “Posttransplantation Lymphoproliferative Disorder of the Paranasal Sinuses Mimicking Invasive Fungal Sinusitis: Case Report,” AJNR. American Journal of Neuroradiology 23, no. 5 (2002): 855–857. 12006293 PMC7974743

