# Supplementary figures and images for: Multisite Head and Neck Pediatric Posttransplant Lymphoproliferative Disorder: A Case Report
Source: Clin Case Rep. 2025 Feb 14;13(2):e70200. doi: 10.1002/ccr3.70200 (PMC11828662; doi:10.1002/ccr3.70200)

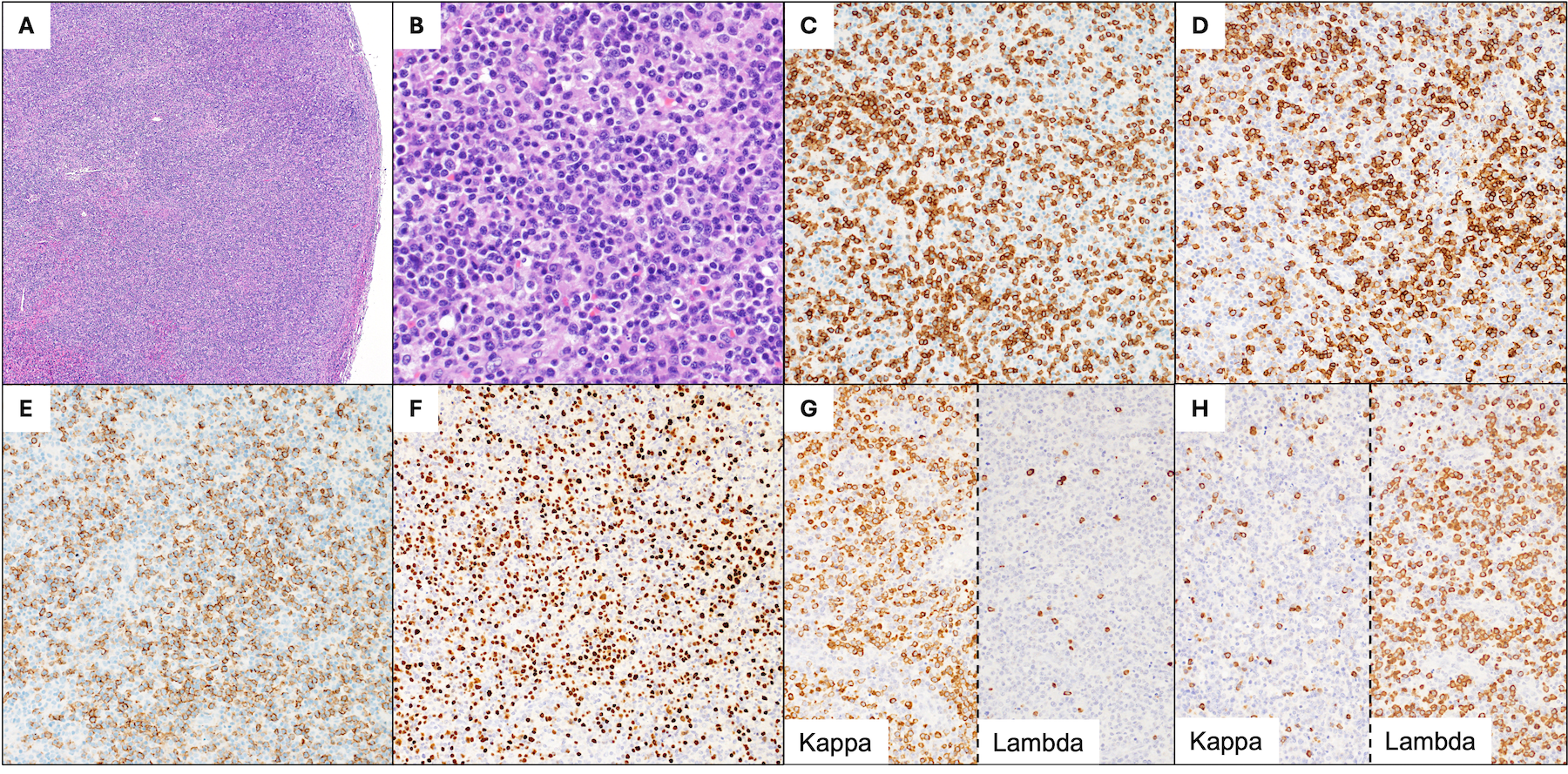

Supplement: Supplementary file 1 — Figure S1. Polymorphic posttransplant lymphoproliferative disorder. (A) Low power (40×) and (B) high power (400×) images show effacement of nodal architecture by a mixed inflammatory infiltrate composed of small to intermediate‐sized lymphocytes, plasma cells, and immunoblasts. Immunohistochemical stains show a mixture of CD3‐positive T cells (C), CD20‐positive B cells (D), and CD138‐positive plasma cells (E). In situ hybridization for EBV‐encoded RNA (EBER) was diffusely positive (F). Plasma cells and plasmacytoid B cells show focal areas with excess kappa light chain expression (G) and other areas with excess lambda light chain expression (H), with polytypic light chain expression overall by kappa/lambda in situ hybridization. All immunostain images 200×. [file CCR3-13-e70200-s001.png]

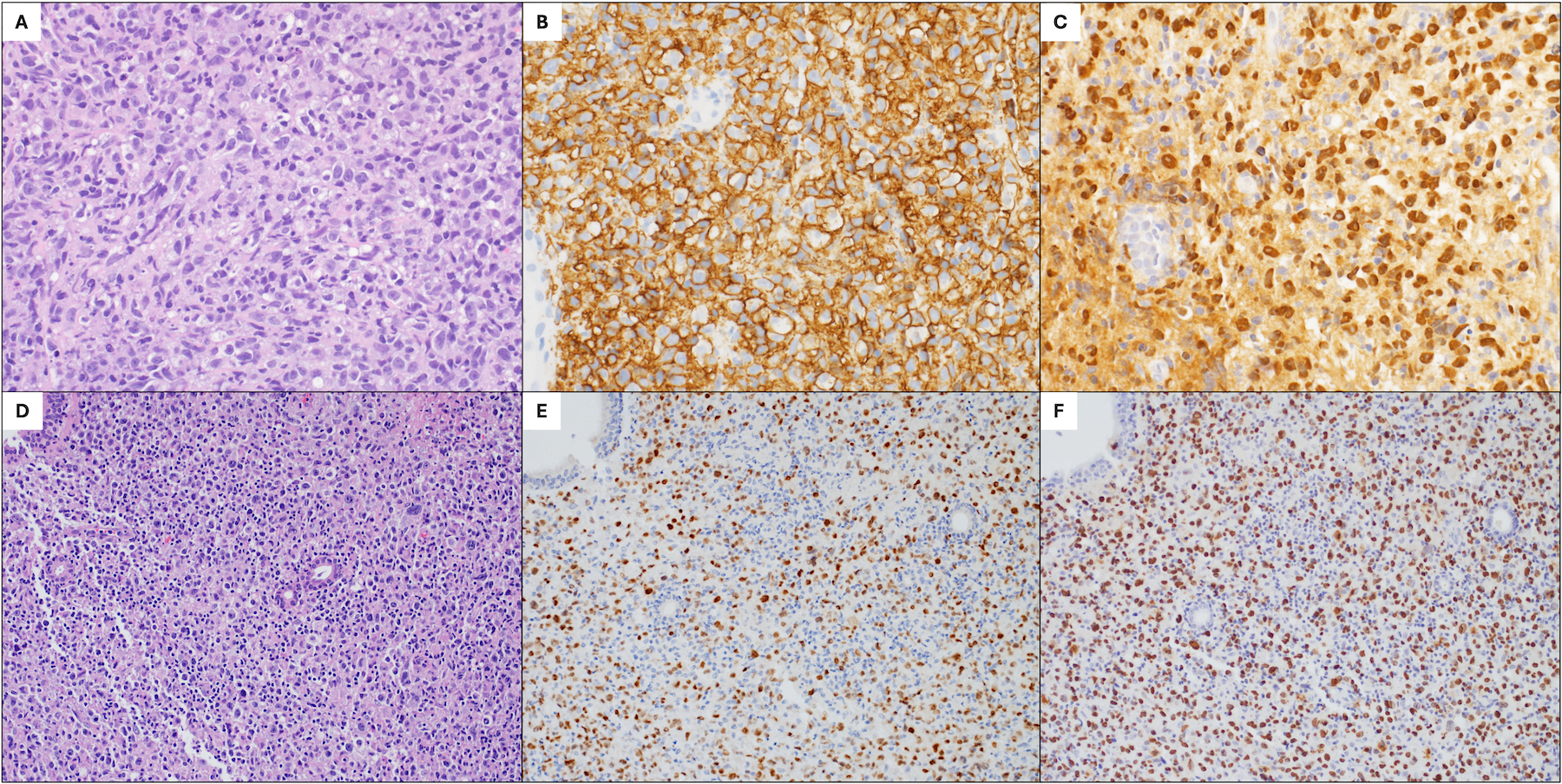

Supplement: Supplementary file 2 — Figure S2. Monomorphic posttransplant lymphoproliferative disorder presenting as EBV‐positive diffuse large B cell lymphoma. (A–C) Biopsies of an umbilicated gastric mass showed a diffuse infiltrate of large lymphocytes with prominent nuclei on H&E stain (A). The large cells were CD20‐positive B cells (B) that were diffusely positive for EBV by EBER in situ hybridization (C). (D–F) Biopsy of the ethmoid sinus showed similar findings on H&E stain (D), with a diffuse infiltrate of large lymphoid cells, which were positive for PAX5 (E) and diffusely positive for EBER (F). (A–C) All images 400×. (D–F) All images 200×. [file CCR3-13-e70200-s002.png]
